# Controversies over the Epithelial-to-Mesenchymal Transition in Liver Fibrosis

**DOI:** 10.3390/jcm5010009

**Published:** 2016-01-14

**Authors:** Kojiro Taura, Keiko Iwaisako, Etsuro Hatano, Shinji Uemoto

**Affiliations:** 1 Department of Surgery, Kyoto University Graduate School of Medicine, Kyoto 606-8507, Japan; etsu@kuhp.kyoto-u.ac.jp (E.H.); uemoto@kuhp.kyoto-u.ac.jp (S.U.); 2 Department of Target Therapy Oncology Kyoto University Graduate School of Medicine, Kyoto 606-8507, Japan; iwaisako@kuhp.kyoto-u.ac.jp

**Keywords:** epithelial-to-mesenchymal transition (EMT), liver fibrosis, myofibroblast, hepatocyte, cholangiocyte

## Abstract

Liver fibrosis is a universal consequence of chronic liver diseases. It is accompanied by activation of collagen-producing myofibroblasts, resulting in excessive deposition of extracellular matrix. The origin of myofibroblasts in the fibrotic liver has not been completely resolved and remains a matter of debate. Recently, the epithelial-to-mesenchymal transition (EMT) was proposed as one of the mechanisms that give rise to collagen-producing myofibroblasts in liver fibrosis. However, subsequent studies contradicted this hypothesis, and the EMT theory has become one of the most controversial and debatable issues in the field of liver fibrosis research. This review will summarize the existing literature on EMT in liver fibrosis and will analyze the causes for the contradictory results to draw a reasonable conclusion based on current knowledge in the field.

## 1. Introduction

Liver fibrosis is the ubiquitous consequence of various chronic liver diseases. These diseases include viral hepatitis (such as hepatitis B virus (HBV) and hepatitis C virus (HCV)), cholestatic liver diseases (such as primary sclerosing cholangitis (PSC), primary biliary cirrhosis (PBC), and congenital biliary atresia (CBA)), alcoholic liver disease (ALD), and non-alcoholic steatohepatitis (NASH) [1]. Liver fibrosis is characterized by a dysregulated wound healing process that results in excessive deposition of extracellular matrix (ECM), primarily collagen type I, and scar formation [2,3]. Activation of collagen-producing myofibroblasts is a fundamental process for the development of liver fibrosis. However, the origin of myofibroblasts in the fibrotic liver has not been fully elucidated and is still under heated debate despite extensive research [4,5]. Recent studies suggested that the epithelial-to-mesenchymal transition (EMT) is one of the mechanisms that gives rise to collagen-producing myofibroblasts in liver fibrosis. However, this hypothesis was contradicted by subsequent studies, and the EMT theory has become one of the most controversial and debatable issues in liver fibrosis research. Here we will review the existing literature on EMT in liver fibrosis and analyze the reasons for the discordant results, aiming to draw a reasonable conclusion based on current knowledge.

## 2. The Sources of Fibrogenic Cells in Liver Fibrosis

Hepatocytes were thought to produce ECM upon fibrotic stimuli until Friedman *et al.* rediscovered hepatic stellate cells (HSCs) (previously known as Ito cells or hepatic lipocytes) as one of the primary sources of collagen-producing cells [6]. This discovery brought HSCs to the center of the liver fibrosis field. HSCs constitute approximately 10% of total liver cells and reside in the space of Disse (the space between hepatocytes and sinusoidal endothelial cells). Under normal physiological conditions, HSCs exhibit a quiescent phenotype, store retinoids in lipid droplets in their cytoplasm and express desmin, glial fibrillar associated protein (GFAP), and nerve growth factor receptor p75. In response to fibrogenic stimuli, HSCs undergo a transdifferentiation process called “activation”, which is characterized by loss of vitamin A-containing lipid droplets, a spindle-shaped morphological change, and expression of α-smooth muscle actin (α-SMA) and ECM represented by collagen type I. The latter two are classical features of myofibroblasts. A recent study by Mederacke *et al.* reevaluated the contribution of HSCs to the myofibroblastic pool in liver fibrosis using modern technology [7]. The group permanently labeled HSCs *in vivo* in mice utilizing lineage tracing (described in detail later) and subjected the mice to various experimental liver fibrosis models such as carbon tetrachloride (CCl_4_) and bile duct ligation (BDL). They found that HSCs gave rise to a majority (82%–96%) of myofibroblasts in the liver irrespective of etiology, and they reconfirmed the central role of HSCs as the origin of liver myofibroblasts. However, their findings also indicated that HSCs were not the only precursor of hepatic myofibroblasts.

The portal fibroblast (PF) is another mesenchymal cell type that resides in the liver and is thought to transdifferentiate into myofibroblasts. Studies have suggested a critical role for PF in cholestatic liver injury, especially at the early stage [8,9,10]. However, PF is not as well-characterized as HSC, due to lack of reliable isolation method and specific markers. Lineage tracing studies showing definitive evidence for the contribution of PF to hepatic myofibroblast populations have not yet been performed, due to lack of a specific PF promoter. Therefore, the extent of the contribution of PF to the myofibroblast pool remains controversial and should be further elucidated in the future.

Another potential source of myofibroblasts in the liver are fibrocytes. Fibrocytes are bone marrow-derived circulating cells that have characteristics of both fibroblasts and hematopoietic cells and are defined by the simultaneous expression of CD45 and collagen type I. CD45^+^Col^+^ fibrocytes migrate to the liver after BDL and comprise 4%–6% of the collagen type I-expressing cells [11]. Similarly, fibrocytes are recruited to CCl_4_-damaged liver, where they differentiate into α-SMA^+^ myofibroblasts *in vivo* [12]. Although the contribution of fibrocytes in liver fibrosis, as well as fibrosis in the other organs, has been established [13,14,15,16], controversy exists as to what extent fibrocytes contribute to the myofibroblast pool in liver fibrosis [17].

The final players that potentially give rise to myofibroblasts in liver fibrosis are epithelial cells, namely, hepatocytes and cholangiocytes. The process by which epithelial cells lose their epithelial characteristics involves apical-basal polarity, intercellular adhesion complexes, and adherence to a basal basement membrane. Additionally, the cells acquire mesenchymal phenotypes, such as motility, migratory ability, and invasiveness. These changes comprise the EMT process and are involved in embryonic development (type 1 EMT). The hypothesis that EMT is also involved in organ fibrosis to yield myofibroblasts is referred to as type 2 EMT, whereas type 3 EMT refers to a process wherein cancer cells lose cell-to-cell contact and acquire invasive capacity. The concept of type 2 EMT was first proposed in renal fibrosis and has taken the fibrosis world by storm. Following renal fibrosis, type 2 EMT was suggested as one of the mechanisms that also gives rise to myofibroblasts in lung fibrosis and liver fibrosis [18,19]. However, the EMT theory in the context of organ fibrosis was seriously questioned by subsequent studies and is perhaps the most intriguing and controversial of recent hypotheses on the mechanism of fibrosis. This review will summarize the published articles for and against EMT in liver fibrosis and will analyze the reasons for the discordant results, with the goal of obtaining reasonable conclusions based on current literature.

## 3. Evidence for Epithelial-to-Mesenchymal Transition (EMT) in Hepatocytes

The first *in vitro* evidence for EMT in hepatocytes was demonstrated by Kaimori *et al.* [20]. This group demonstrated that hepatocytes treated with transforming growth factor β-1 (TGFβ-1) underwent a phenotypic change and gene expression pattern leading to mesenchymal cell features. Specifically, the phenotypic change was a spindle-shaped morphology with polarization of F-actin stress fibers. The changes in gene expression included loss of albumin, downregulation of the epithelial marker E-cadherin, and upregulation of the mesenchymal marker vimentin. Interestingly, fibroblast-specific protein-1 (FSP-1), a marker widely used to identify cells undergoing EMT, was not upregulated after TGFβ-1 treatment in hepatocytes in their study. This latter finding was in sharp contrast to Zeisberg’s study, which demonstrated co-staining for albumin and FSP-1 in TGFβ-1-treated primary hepatocytes [21]. Although the reason for the discrepancy between the two studies is unknown, it should be noted that *in vitro* data supporting EMT is derived from a highly artificial condition in which there is no vascular, endocrine, or neurologic contribution and that does not recapitulate the complexity of real tissue. Nitta *et al.* demonstrated not only that primary hepatocytes undergo EMT in response to TGFβ-1 but also that primary hepatocytes from CCl_4_-induced cirrhotic livers exhibit features of EMT even without TGFβ-1 treatment in culture [22]. Their observation implied that EMT in this context is not a mere artifact of an *in vitro* experiment but may occur *in vivo*.

Showing direct evidence of EMT *in vivo* is challenging. Because EMT is a transition from epithelial cells to mesenchymal cells, cells that are in the midst of EMT are thought to express both epithelial and mesenchymal markers. Therefore, co-expression of epithelial and mesenchymal markers is often used as a criterion to identify epithelial cells that are undergoing EMT. Common epithelial markers include E-cadherin and cytokeratin, whereas typical mesenchymal markers include N-cadherin, α-SMA, collagen 1α1, vimentin, and the most frequently used marker in the field of EMT, FSP-1 (also known as S100A4). Zeisberg *et al.* induced liver fibrosis in mice with CCl_4_ and stained liver sections for albumin (a hepatocyte marker) and FSP-1 (a mesenchymal marker) and observed albumin-FSP-1 double positive cells [21]. Nitta *et al.* demonstrated vimentin-positive hepatocytes in cirrhotic liver in mice [22]. Although this simple technique is useful for identifying cells that are presumably undergoing EMT, there are technical limitations to be kept in mind. Immunostaining itself is an experimental procedure that may cause many nonspecific signals. Damaged tissues in particular tend to adsorb antibodies in a nonspecific manner, which results in nonspecific staining. Some mesenchymal markers may not be expressed by all types of mesenchymal cells, which limits the sensitivity of this approach. On the other hand, some mesenchymal markers may not be specific, limiting the specificity of this technique. In addition, an epithelial cell undergoing EMT may have not yet fully activated mesenchymal gene expression, and experiments could therefore miss cells at the beginning of the EMT process. Moreover, even when staining suggests that an epithelial marker and a mesenchymal marker colocalize, it is often difficult to conclude that a single cell actually expresses the two markers because it is difficult to fully exclude the possibility that an adjacent or adherent cell is actually expressing one of the markers. Therefore, although identifying EMT based on marker expression is a convenient and powerful tool, as well as virtually the only methodology applicable to human studies, we should be very cautious about interpreting the data from these methods and look to more sophisticated experimental approaches for firmer evidence of *in vivo* EMT.

To overcome the limitations of the colocalization technique, so-called ‘‘lineage tracing’’ or ‘‘fate mapping’’ was used to prove EMT *in vivo*. This technology is designed to label a specific cell population, which enables investigators to track the fate of the labeled cells. This strategy utilizes cell type-specific activation of gene regulatory elements to permanently label the cells with a reporter gene (e.g., *LacZ*) [23]. For example, transgenic mice have been created in which Cre recombinase is expressed under control of the albumin promoter (Alb-Cre mice). In these animals, Cre recombinase is expressed in the cells that activate the albumin gene promoter, that is, hepatocytes. Cre recombinase is an enzyme that cleaves loxP sites, removing “floxed” segments of DNA flanked by those loxP sites. When Alb-Cre mice are bred with another transgenic mouse with floxStopfloxLacZ, the stop repressor is excised only in the cells expressing Cre recombinase, namely, hepatocytes. This alteration occurs at the DNA (genome) level and is passed to progeny. Therefore, all progeny of the cells that had an activated albumin promoter at some point are permanently tagged by LacZ, resulting in marking of all hepatocyte-derived cells regardless of differentiation, proliferation, and migration in the context of tissue injury.

Zeisberg *et al.* were the first to utilize this methodology to prove the concept of EMT in liver fibrosis [21]. They generated double transgenic mice Alb-Cre x ROSA26 floxStopfloxLacZ, in which hepatocyte-derived cells expressed β-galactosidase, as described above. These mice were then subjected to CCl_4_ treatment to induce liver fibrosis, and the investigators examined whether hepatocyte-derived fibroblasts existed. They utilized FSP-1 as a marker for fibroblasts and demonstrated colocalization of β-galactosidase and FSP-1 on liver sections by immunofluorescence. They also demonstrated that primary hepatocytes from this double transgenic mouse became positive for both FSP-1 and β-galactosidase in response to TGFβ-1 treatment, excluding the possibility that EMT observed in primary hepatocytes was a consequence of contaminating cells. These findings have been widely cited and were generally considered to provide the most definitive evidence for the concept that hepatocyte EMT contributes to the fibrogenic cell source in liver fibrosis.

## 4. Evidence against Hepatocyte EMT

Studies supporting the concept of EMT were seriously challenged by later work using lineage tracing. Taura *et al.* published an elaborate study in which double transgenic mice Alb-Cre x ROSA26 floxStopfloxLacZ were bred with transgenic mice expressing green fluorescent protein (GFP) under control of the collagen 1α1 promoter (Coll GFP mice) [24] to generate triple transgenic mice in which β-galactosidase was expressed in “hepatocyte-derived” cells and GFP was expressed in “collagen-expressing” cells [25]. The authors did not detect a transition of Lac Z-positive (hepatocyte-derived) cells into GFP-positive (collagen-expressing) myofibroblasts in CCl_4_-induced fibrotic liver. They confirmed this essential finding in three different ways: (1) Overlaying images of GFP fluorescence onto images of X-gal-stained liver sections demonstrated no colocalization of GFP-positive cells and X-gal-positive cells; (2) Whole liver cells were isolated from CCl_4_-treated liver and no cells were double positive for GFP and X-gal; and (3) GFP-positive cells were isolated from CCl_4_-treated liver by fluorescent activated cell sorting (FACS) and no cells were positive for X-gal. The consistent results from these experiments strongly questioned the contribution of hepatocytes to a pool of collagen-producing fibroblasts in the liver. Another intriguing result from the same work was the *in vitro* experiment. The authors isolated hepatocytes from the triple transgenic mice and treated them with TGFβ-1. The hepatocytes exhibited the spindle-shaped morphological change compatible with EMT and expressed GFP, indicating activation of the collagen 1α1 promoter. However, these alterations were not accompanied by activation of mesenchymal genes, such as α-SMA or FSP-1. These findings indicated that their observations *in vitro* did not fulfill the criteria for EMT but could be an artifact in experiments in culture dishes. However, the significance of the morphological change and the driving of the collagen promoter remained a matter of interest that should be addressed in the future.

Another strong piece of evidence against hepatocyte EMT was provided by Chu *et al.* [26]. They utilized α-fetoprotein-Cre (AFP-Cre) mice to label any cells that expressed AFP at any point, namely, hepatocytes, cholangiocytes, and their bipotential progenitor cells. The mice were crossed with ROSA26 floxStopflox yellow fluorescent protein (YFP) mice to generate double transgenic mice in which all the epithelial cells, bipotential progenitor cells, and their progeny in the liver were tagged with YFP. This group developed three elaborate experimental liver fibrosis models (CCl_4_, BDL, and 3,5-diethoxycarbonyl-1,4-dihydrocollidine (DDC)), and in no case did they find evidence of colocalization of YFP with the mesenchymal markers FSP-1, vimentin, α-SMA, or pro-collagen 1α2. Compared with the preceding work by Taura *et al.* [25] utilizing Alb-Cre mice to tag hepatocytes, the marker efficiency appeared higher, challenging the criticism that non-tagged hepatocytes that had not fully activated the albumin promoter actually underwent EMT but escaped detection. Similar to what had been observed in primary hepatocytes in the study by Taura *et al.* [25], primary cholangiocytes showed a morphological change consistent with EMT in response to TGFβ-1 in the work by Chu, further emphasizing that observations from *in vitro* experiments often do not translate into *in vivo* settings.

## 5. Why Did Studies on Hepatocyte EMT Reach Such Different Conclusions?

Reconciling all of the conflicting data mentioned above is not easy. The studies referenced adopted similar lineage tracing techniques and the same experimental liver fibrosis model (CCl_4_-induced liver fibrosis) and yet reached opposite conclusions, making interpretation extremely difficult. Closely examining the results of each of the studies will provide clues to draw a reasonable conclusion for the time being.

### 5.1. Questioning the Role of Fibroblast-Specific Protein-1 (FSP-1) as a Marker for Fibroblasts and Cells Undergoing EMT

The most debatable issue in the study by Zeisberg was the sole use of FSP-1 as a marker to detect myofibroblasts. FSP-1 was first described by Strutz *et al.* in 1995 as a murine fibroblast-specific protein belonging to the calmodulin-S100-troponin C superfamily of intracellular calcium-binding proteins [27]. Transfection of FSP-1 into non-metastatic cells induced a migratory or metastatic capacity, which suggested that FSP-1 was associated with a mesenchymal phenotype [28]. Tubular epithelial cells in the kidney showed features of EMT, such as loss of cell adhesion and expression of vimentin upon transfection with FSP-1 [27]. Due to these properties, FSP-1 was regarded as a valid marker for fibroblasts and cells undergoing EMT [29]. However, detailed studies on the distribution and role of FSP-1 positive cells in other organs, including the liver, were lacking. Moreover, even in the kidney, where the functional role of FSP-1 was originally identified, the validity of FSP-1 as a marker for EMT was questioned. Careful examination of the distribution of FSP-1-positive cells in the kidney suggested that FSP-1 was a marker, not of fibroblasts, but rather of leukocytes and other, non-fibroblastic cell types [30]. In the liver, extensive work to characterize FSP-1-positive cells was performed by Österreicher *et al.* [31]. This group observed increased numbers of FSP-1-positive cells in fibrotic livers. The FSP-1-positive cells were distributed mainly along fibrotic septa; however, they were not fibroblasts, as evidenced by a lack of GFP expression in FSP-1-positive cells in Coll GFP mice subjected to BDL or CCl_4_. Then, FSP-1 GFP mice, in which GFP was expressed under FSP-1 promoter, were subjected to liver injury models. Again, GFP-positive cells did not express α-SMA or desmin, typical markers of activated myofibroblasts. The authors carefully examined the possibility that FSP-1-positive cells were the precursor of myofibroblasts that had not yet fully activated mesenchymal gene expression. They did so through a lineage tracing study with double transgenic mice FSP-1-Cre x ROSA26 floxStopfloxYFP, in which FSP-1-positive cells were permanently labeled with YFP. As before, they did not find YFP-positive cells with α-SMA or desmin expression in injured liver, indicating that FSP-1-positive cells are neither myofibroblasts nor their precursors. Instead, gene profiling analyses characterized FSP-1-positive cells as having a myeloid-monocytic lineage, as suggested by expression of F4/80 and other markers. The role of FSP-1 as a marker for EMT in the fibrotic liver was seriously questioned by these findings, which also emphasized the importance of finding more appropriate markers based on the fundamentals of active myofibroblasts to identify cells undergoing EMT.

### 5.2. Unreliability of Immunofluorescence Studies for the Detection of β-Galactosidase

Another issue to be carefully examined in the study by Zeisberg [21] is the reliability of immunofluorescence studies to detect β-galactosidase expression. The authors employed both X-gal staining and immunofluorescence; however, to demonstrate colocalization with FSP-1, they exclusively used immunofluorescence. Their approach seemed reasonable, as the double immunofluorescence technique is a convenient way to demonstrate double positivity for two different markers. In our experience, however, β-galactosidase expression driven by the ROSA26 promoter is not high enough to be detected by immunofluorescence with anti-β-galactosidase antibodies. We carefully conducted control experiments to confirm β-galactosidase activity in the transgenic mice used in the Zeisberg study (and in our study [25] as well). We found that the activity was much lower than that induced by an adenoviral vector encoding β-galactosidase, as suggested by the difference in the incubation time required to obtain blue staining from the X-gal method between the transgenic mice (overnight) and those treated with adenovirus (a few hours). Immunofluorescence worked for adenovirally-expressed β-galactosidase, thereby excluding the possibility that the antibody we used was of poor quality. From these preliminary experiments, we reached the conclusion that X-gal staining, not immunofluorescence, should be the method of choice for detecting β-galactosidase expression in the transgenic mice, and we subsequently used this method exclusively. Duffield also supported our suggestion that immunofluorescence for β-galactosidase is unreliable [32]. We, therefore, suppose that the immunofluorescence for β-galactosidase used by Zeisberg was not reliable enough to prove colocalization with FSP-1. Rather, it could represent nonspecific staining, which is suggested by the following observations: (1) In liver sections, the staining pattern of immunofluorescence was diffuse and appeared different from the staining pattern of X-gal, which was more spotty and patchy; and (2) In isolated cells, the staining patterns of the two different antigens, namely β-galactosidase and FSP-1, were nearly identical, suggesting nonspecific staining or bleed-through from another fluorescent probe.

## 6. Evidence Suggesting Cholangiocyte EMT

Cholangiocytes are another type of epithelial cell in the liver. With the attention given to hepatocyte EMT in liver fibrosis, it became obvious that investigating whether cholangiocytes underwent EMT and contributed to the fibrogenic cell pool should be the next focus. Indeed, several studies supported the concept of cholangiocyte EMT. Omenetti *et al.* induced biliary fibrosis in rats by BDL and demonstrated that isolated cholangiocytes upregulated expression of FSP-1 (a mesenchymal marker) and downregulated expression of aquaporin-1, cytokeratin 7, and cytokeratin 19 (epithelial markers) [33]. In another study, they also demonstrated that the Hedgehog signaling pathway, which was considered the key signaling pathway inducing EMT, was activated in the biliary epithelium in BDL mice [34]. In addition, an immature cholangiocyte line cultured with conditioned medium from activated (myofibroblastic) hepatic stellate cells was shown to undergo EMT, demonstrated by reduced expression of epithelial markers, induction of mesenchymal markers, and acquisition of a migratory phenotype [33]. Omenetti *et al.* further documented blockade of Hedgehog signaling by a Hedgehog ligand-neutralizing antibody that suppressed EMT in the cholangiocytes treated with conditioned medium from activated hepatic stellate cells [35]. Taken together, this work provided good evidence indicating that cholangiocytes were capable of EMT in animal models. Furthermore, human studies also suggested the presence of cholangiocyte EMT in various cholestatic diseases. For example, Rygiel *et al.* demonstrated co-expression of epithelial and mesenchymal markers in biliary epithelial cells in liver sections from PBC and PSC [36]. Diaz *et al.* observed colocalization of CK19 (a biliary epithelial cell marker) and mesenchymal markers such as FSP-1 and vimentin in cholangiocytes in patients with biliary atresia and PBC [37]. However, evidence derived from the aforementioned studies was based exclusively on the “colocalization technique” or “*in vitro*” observations and direct genetic evidence for conversion of cholangiocytes to myofibroblasts *in vivo* was lacking.

## 7. No Evidence of Cholangiocyte EMT by Lineage Tracing

To investigate whether cholangiocytes contribute to the myofibroblastic pool in hepatic fibrosis, lineage tracing was employed, as it was to demonstrate the concept of hepatocyte EMT. Scholten *et al.* labeled cholangiocytes with YFP by crossing ROSA26 floxStopflox YFP with tamoxifen-inducible CK19-CreERT [38]. The use of inducible CK19-Cre was necessary to avoid effects of CK19 activation during development [39]. These double transgenic mice were subjected to BDL, a cholestatic liver injury model that most likely involves cholangiocyte EMT. As a result, the authors found that none of the YFP-positive cholangiocytes upregulated myofibroblast markers such as α-SMA and FSP-1 in livers of BDL-injured mice. As mentioned in the hepatocyte EMT section above, another study by Chu *et al.* [26] found no evidence that AFP-positive cells, namely bipotential epithelial progenitors (hepatocytes and cholangiocytes), gave rise to the myofibroblastic cell population, further supporting the absence of cholangiocyte and hepatocyte EMT. Similar to the argument over hepatocyte EMT, advanced genetic technology refuted cholangiocyte EMT.

## 8. Controversies over EMT in Fibrosis of Other Organs

Reviewing the debates on EMT in other organs might help us reach a reasonable conclusion. EMT in the context of organ fibrosis was first described in the kidney. Iwano *et al.* first utilized lineage tracing to demonstrate that renal epithelial cells give rise to myofibroblasts [40]. They labeled renal epithelial cells by crossing γGT Cre mice with ROSA26 floxStopfloxLacZ mice. The mice were subjected to unilateral ureteral obstruction (UUO) to induce renal fibrosis, resulting in the appearance of cells double positive for β-galactosidase and FSP-1 by immunofluorescence. However, similar to the story of EMT in liver fibrosis, the EMT concept in renal fibrosis was challenged by several lineage tracing studies published thereafter. Humphreys *et al.* labeled all renal epithelial cells in kidney using two separate and well-characterized developmentally active Cre-driver lines: the Six2-cre driver, which labels metanephric mesenchyme-derived epithelia [41], and the HoxB7-cre driver, which labels ureteric bud-derived collecting duct epithelia [42]. These mice were crossed with reporter lines and the double transgenic offspring were subjected to either UUO or ischemia-reperfusion injury to induce renal fibrosis. From these experiments, the authors found no evidence of renal epithelial cell-derived α-SMA-positive myofibroblasts. Since publication of the above studies, several other studies utilizing lineage tracing in renal fibrosis have been reported that also argued against the EMT hypothesis [43,44,45]. Grgic *et al.* raised doubt on the immunofluorescence-based detection of β-galactosidase in Iwano’s study and speculated that the discordant findings in Iwano’s study was caused by the false-positivity of β-galactosidase detection. We would also note that another methodological concern about Iwano’s study was its heavy reliance on FSP-1 for the identification of myofibroblasts [30]. Taken together, these papers show that the discussion on the presence or absence of EMT in renal fibrosis was just like the debate surrounding EMT in liver fibrosis.

Lung fibrosis is another field where controversy around the contribution of EMT is heated. Indeed, more fate mapping studies supporting EMT have been published in lung fibrosis than in liver or renal fibrosis [46,47,48,49,50]. However, a more recent study by Rock *et al.* argued against EMT in lung fibrosis [51]. In this study, the authors criticized use of the enzymatic reaction using X-gal staining for β-galactosidase detection as it does not allow high-resolution confocal imaging, unlike fluorescent tags. As a result, the borders between neighboring cells became obscure and the possibility of false-positive detection of cells double-positive for myofibroblast markers and β-galactosidase cannot be excluded.

## 9. Provisional Conclusions and Future Perspective

It should be acknowledged that recent studies against the EMT concept in liver fibrosis also have methodological concerns. First, like all other experimental techniques, lineage tracing has some pitfalls. The efficiency of Cre-mediated recombination is not 100%. For instance, in the study by Scholten *et al.*, efficacy of Cre-recombination in their CK19-Cre/YFP mice was approximately 40% [38]. Our group also observed a decreasing proportion of labeled hepatocytes in Alb-Cre/LacZ mice as the number of CCl_4_ injections increased. Therefore, it is theoretically possible that EMT might have occurred in the non-labeled cells, although this is unlikely unless EMT occurs selectively in cells where Cre-mediated recombination does not take place. Second, most lineage tracing studies still rely heavily on immunostaining to determine whether a cell acquires a mesenchymal phenotype. The use of only a few mesenchymal markers might not be sufficient to completely rule out the existence of EMT. It is also possible that expression level of some markers are not strong enough to be detected by current immunostaining techniques. Finally, experimental liver fibrosis models are different from the pathophysiological process that occurs in human liver diseases in terms of length or strength of fibrotic stimuli. Therefore, these experimental models may not truly reflect pathophysiological events in human chronic liver diseases and therefore cannot completely exclude the possibility of EMT in humans. However, regardless of the above-mentioned criticisms of the studies indicating a lack of EMT in liver fibrosis, analysis of the reasons for the discordant results effectively refuted published data that supported the EMT concept in liver fibrosis. Therefore, we should not now stand behind the assumption that EMT does occur and gives rise to myofibroblasts in liver fibrosis. Instead, we have to return to the presumption that EMT does not contribute to liver fibrosis. It is obvious that new lines of evidence will be required for EMT to reemerge as a viable concept in liver fibrosis. Unless a new evidence for EMT is established, it seems reasonable that antifibrotic therapies should target HSCs, as has been done for decades.

## Abbreviations

The following abbreviations are used in this manuscript:

AFP: α-fetoprotein
ALD: alcoholic liver disease
α-SMA: α-smooth muscle actin
BDL: bile duct ligation
CBA: congenital biliary atresia
CCl_4_: carbon tetrachloride
ECM: extracellular matrix
EMT: epithelial-to-mesenchymal transition
FACS: fluorescent activated cell sorting
FSP-1: fibroblast-specific protein 1
GFAP: glial fibrillar associated protein
HBV: hepatitis B virus
HCV: hepatitis V virus
HSC: hepatic stellate cell
NASH: non-alcoholic steatohepatitis
PBC: primary biliary cirrhosis
PF: Portal fibroblast
PSC: primary sclerosing cholangitis
TGFβ-1: transforming growth factor β-1
UUO: unilateral ureteral obstruction
